# Applications of Polymers for Organ-on-Chip Technology in Urology

**DOI:** 10.3390/polym14091668

**Published:** 2022-04-20

**Authors:** Bianca Galateanu, Ariana Hudita, Elena Iuliana Biru, Horia Iovu, Catalin Zaharia, Eliza Simsensohn, Marieta Costache, Razvan-Cosmin Petca, Viorel Jinga

**Affiliations:** 1 Department of Biochemistry and Molecular Biology, University of Bucharest, 91-95 Splaiul Independentei Street, 050095 Bucharest, Romania; bianca.galateanu@bio.unibuc.ro (B.G.); marieta.costache@bio.unibuc.ro (M.C.); 2 Advanced Polymer Materials Group, Department of Bioresources and Polymer Science, University Politehnica of Bucharest, 1-7 Gh. Polizu Street, 011061 Bucharest, Romania; horia.iovu@upb.ro (H.I.); catalin.zaharia@upb.ro (C.Z.); 3 Academy of Romanian Scientists, Ilfov Street, 50044 Bucharest, Romania; 4 “Carol Davila” University of Medicine and Pharmacy Bucharest, 050474 Bucharest, Romania; eliza.simsensohn@gmail.com (E.S.); razvan.petca@umfcd.ro (R.-C.P.); viorel.jinga@umfcd.ro (V.J.)

**Keywords:** organ-on-chip, tumor-on-chip, polymeric microfluidic devices, kidney-on-chip, bladder-on-chip, prostate-on-chip

## Abstract

Organ-on-chips (OOCs) are microfluidic devices used for creating physiological organ biomimetic systems. OOC technology brings numerous advantages in the current landscape of preclinical models, capable of recapitulating the multicellular assemblage, tissue–tissue interaction, and replicating numerous human pathologies. Moreover, in cancer research, OOCs emulate the 3D hierarchical complexity of in vivo tumors and mimic the tumor microenvironment, being a practical cost-efficient solution for tumor-growth investigation and anticancer drug screening. OOCs are compact and easy-to-use microphysiological functional units that recapitulate the native function and the mechanical strain that the cells experience in the human bodies, allowing the development of a wide range of applications such as disease modeling or even the development of diagnostic devices. In this context, the current work aims to review the scientific literature in the field of microfluidic devices designed for urology applications in terms of OOC fabrication (principles of manufacture and materials used), development of kidney-on-chip models for drug-toxicity screening and kidney tumors modeling, bladder-on-chip models for urinary tract infections and bladder cancer modeling and prostate-on-chip models for prostate cancer modeling.

## 1. Introduction

Regardless of the therapeutic area of new emerging therapies and novel agents entering the market, nephrotoxicity is a major challenge, as the kidney is the second target of drugs and chemicals after the liver. More than 25% of the adverse effects in today’s pharmacotherapy are caused by nephrotoxic effects [1]. Of these, 20% are reported during postmarket surveillance [2], as early stages of drug development fail to deliver relevant output with this respect [3]. Consequently, the poor correlation between the preclinical and clinical outcomes has led to the failure of most drugs before reaching the patient [4]. Preclinically approved drugs have been withdrawn a few times due to the side effects observed in the clinical trials [5,6]. Therefore, the pharmaceutical industry is under high pressure to speed up the drug-development process and to design new cures that are very effective in humans with reasonable costs [7].

The traditional in vivo tests on animal models are costly and often fail to accurately predict the efficiency and toxicity in humans due to the species’ different metabolic responses to specific agents and the variations in some genes’ expressions, such as cytochrome P450 genes [8]. Consequently, the poor similarity between the physiological environment in animals and human bodies, which may alter the results of drug efficiency in various diseases, is a major barrier for future use of in vivo tests on animals [9]. With respect to cancer research, animal models in particular lack predictability [10] since they do not recreate the exact human tumor microenvironment (TME) and may exhibit different cell biology and cancer behavior when tumorous cells interact locally with stromal cells. In addition, the ethics concerns of sacrificing animals are a significant barrier in testing many discovered drugs on animals [11,12]. In 2021, the European Parliament agreed with a large majority to ban experiments on animals, which have killed about 12 million animals in 2017, revealing the importance of finding alternatives for biological assays developed with other procedures than sacrificing animals [13].

Despite tremendous research efforts and advanced medical treatments, cancer continues to be one of the most frequent causes of death in the world. In 2020, 18.1 million new cases and 9.5 million cancer-associated deaths were reported worldwide [14]. According to American Institute for Cancer Research, more than 50% of cancer cases were reported in men. Prostate, bladder, and kidney cancers account for an estimated 23% of all cancers diagnosed in men in the last two years. Furthermore, recent World Health Organization (WHO) statistics are estimating a ~55% increase of overall cancer cases from 2020 to 2040 [15]. Alarmingly, the mortality rate is expected to increase by ~65% by 2040, highlighting the urge to find and acquire more efficient anticancer remedies.

The main difficulties in cancer research are forming an effective in vitro TME able to accurately recapitulate the local tissue in which the tumor is forming [16]. Conventional preclinical in vitro models for anticancer drug screening are generally classified in 2D cell cultures and 3D cell architectures and have been extensively exploited as simple and cost-efficient methods to simulate cancer propagation and drug response [17]. The 3D cancer models deliver a helpful substitute to animals, but they still do not consider the dynamic environment of the human tissues or organs. However, they do not reproduce the complex assemblage of the human 3D cells from living organs to properly elucidate the cancer cell migration and invasion, also taking into consideration the mechanical forces (such as hydrostatic pressure, fluid shear stress, breathing motions in lung) naturally occurring in human bodies. Nonetheless, neither of these systems is transporting a blood or nutrient-rich medium through an endothelium-lined vasculature, limiting the real prediction of tissue–tissue interactions and circulating immune cells during therapeutic drug dosage [18,19].

Standard cell-culture techniques fail to provide insights into complex multifaced interactions that take place in a multiorgan system. The need to transport fluids containing pharmaceutical compounds through models of different pathological conditions while accurately simulating physiological processes is challenging the scientific media to engineer new technology able to replace animal testing.

Microfluidics is the science and technology of systems that allow the processing and manipulation of microscale fluids (10^−9^ to 10^−18^ L) using channels with sizes of tens to hundreds of micrometers [20]. By combining microelectronics with structural analysis and molecular biology, microfluidics leads to a deeper understanding of the mechanism by which the cellular, biochemical, and physiochemical environment indicate tumor sensitivity and resistance to therapy [21].

Recently, new devices known as organ-on-chips (OOCs), which are able to recapitulate the multicellular assemblage, tissue–tissue interactions, and to replicate human pathologies and the appropriate physical TME, have emerged as a practical cost-efficient solution for tumor-growth investigation and anticancer-drug screening by combining the microfluidic technology with 3D cell-culture procedure to simulate the entanglement of the cells as in their native environment [19,22,23]. OOCs are compact and easy-to-use microphysiological functional units that recapitulate the native function and the mechanical strain that the cells experience in the human bodies, allowing the development of a wide range of applications such as disease modeling or even the development of diagnostic devices. However, important features of the membranes involved in the fabrication of OOC compartments to allow cells’ structural support and nutrient transportation are often poorly investigated. Nowadays, both synthetic and natural polymers are explored for the manufacturing process of advanced OOC microdevices, being able to replicate various organ bionic pathophysiological models. Poly(dimethylsiloxane) is one of the most employed synthetic polymers used for lung, liver, heart, and multi-organ-on-chip (MOOC) membranes in microfluidic devices due to its extraordinary high transparency and flexibility. However, it is not a degradable material able to contribute to the formation of the natural extracellular matrix (ECM). Alternative biopolymers with higher biocompatibility, such as collagen-based materials containing cell-growth factors and hormones, have been used for OOC fabrication to simulate the physiological behavior of living organs. Despite significant advances, many polymeric materials still do not meet the mechanical properties of the in vivo organs and do not exhibit optimal cytocompatibility suitable for accurate pharmaceutical screening or dynamic simulation of cancer cell behavior.

This review brings to attention the specifications and fabrication methods for OOCs and the importance of polymeric porous materials used in OOCs in relation to cell behavior. In this context, the current work aims to review the scientific literature in the field of microfluidic devices and materials designed for urology applications in terms of OOC fabrication (principles of manufacture and materials used), development of kidney-on-chip models for drugs toxicity screening, and kidney tumors modeling, bladder-on-chip models for urinary tract infections and bladder cancer modeling, and prostate-on-chip models for prostate cancer modeling.

## 2. Fabrication of Organ-on-Chip

### 2.1. Principle and Manufacture of Organ-on-Chips

Microfluidic technologies have rapidly developed in the past years in terms of fabrication methodology, materials involved, and complexity of the systems to faithfully respond to the medical requirements. OOCs are microfluidic cell-culture micromachines that can recapitulate an organ-level response to medical treatment and reconstruct physiological dynamics observed in native human tissues, for instance, physiological flow, biomechanical motions, nutrient transportation, and drug delivery [22], in a convenient manner, delivering a platform with a new opportunity for oncology research. As biology-inspired engineered microdevices [24], the OOCs for tumor investigation must enable a series of possibilities: (i) the introduction of pharmaceutical compounds or reagents as fluids with the similar dynamic flow as for biological fluids; (ii) the ability to perfuse these fluids around on the chip, and to combine and mix them; (iii) the introduction of other sensors or devices for monitoring the results, such as detectors for bioanalysis.

By incorporating tumor organoids in microfluidic devices, “tumor-on-chip” (TOCs) models that allow the reconstruction of the TME are created. These chips enable a deeper understanding of the tumor mechanism in vivo, which runs to enhanced preclinical evaluation of drug efficiency. Human solid tumors are highly heterogeneous [16], owning a complex microenvironment with a dense ECM, abnormal vessels, various stromal cells, or different immune-type cells [22]. Additionally, the nearby stromal tissues of the tumor act as an active source (and reservoir) of different cytokines and growth factors that affect the tumor development and pharmacological feedback. Several studies have shown that the complex variety of the cellular microenvironment may impact in some respect the tumor behavior, including tumorigenesis, angiogenesis, tumor invasion, metastasis, and endurance to therapeutic products. Many compounds generated by tumorous or stromal cells determine the propagation dynamics of solid tumors. Moreover, the 3D nature and the size of the tumor proved to be an utmost concern in the proper understanding of tumor dynamics showing a direct connection between the size of a tumor and its aggressiveness and the ability of a drug to be delivered to it [25,26,27]. Consequently, cancer is a true suite of complex pathologies that share some common elements and presents few differences that seriously complicate the choice of satisfactory treatment. Unlike in vivo tumor-growth microenvironments, in vitro cancer models are typically investigated under atmospheric conditions not specific to the living organs. In an in vitro metastasis cell culture, migrated tumor cells have to be subjected to varying microenvironments and oxygen gradients to mimic the activity of in vivo intravasation [28]. Tumor chips combine micromachining and cell biology to manipulate the external conditions and precisely mimic physiological environments, such as dynamic mechanical stress, fluid shear, oxygen, and drug concentration gradients and cell patterning to reflect the full picture of tumor formation and growth mechanism [29,30].

As TOCs resulted from the need to investigate appropriately cancerous cells in their specific TME, the fabrication technology is the same as for conventional OOCs, whereas the healthy organ cells were replaced by diseased cells. The microdevices are named chips since they were originally engineered using micromanufacturing techniques used in computer-microchip production [20]. Microfluidic systems can be employed to form tumor chips with a single-line channel by engaging cells from a single source, or more complex organ chips that associate two or more tissue categories that can be interposed right through a porous ECM-coated membrane or an ECM gel that fills one or more micronetworks [31]. The viability of the cells can be preserved over prolonged time intervals (up to several months) by perfusing the culture medium either across the endothelium-lined vascular microchannels, parenchymal microchannels, or both. More importantly, the culture medium can be substituted by blood for several hours through endothelialized vascular channels [32].

There are two steps required to be taken into consideration for the proper design of a tumor chip: (1) to comprehend the fundamentals essential for the physiological function of the aimed organ, and afterward to establish the key factors such as various cell types, structures, and the organ’s particular physiological microenvironment; (2) to construct a cell-culture device relying on the identified features. Different procedures have been embraced to build tumor-chip kits, among which the most extensively engaged are photolithography and soft lithography [33,34], replica molding [35], microcontact printing, and bioprinting techniques [36,37,38].

#### 2.1.1. Lithography

Lithography represents an etching process applied in microfabrication to project parts on a thin layer or the form of a substrate (also named a wafer) and is commonly divided into three categories: photolithography (or UV lithography), soft lithography, and replica molding. Photolithography employs light to transfer a geometric shape from a photomask to a photosensitive chemical photoresist on the wafer. In the first step, masks are required that correspond to the targeted constructions [22,34]. The manufacturing process continues with the deposition of a spin-coated photoresist layer on a wafer that can be corroded by chemical reagents, for instance, silicon, glass, or quartz, and the photoresist is subjected to UV photopolymerization. Afterward, the pattern is moved to the wafer and is etched to achieve a microfluidic chip with microflow channels. Although extensively employed, the lithography-manufacturing processes are costly due to the cleanroom requirements, the necessity of multiple masks, and time-consuming multiple processing steps. Recently, Kasi and coworkers proposed a rapid-prototyping organ-chip device using maskless photolithography [39]. The authors reported a simplified method that describes a rapid and cleanroom-free microfabrication compatible with soft lithography for fast-prototyping organ-chip devices in a maximum of 8 h. Soft lithography used for tumor-chip microfabrication involves in the first stage the preparation of a microchannel mold on a silicon substrate by photolithography [40], followed by the use of a liquid polymer (commonly polydimethylsiloxane, PDMS) to discharge the mold to acquire an optically transparent elastomeric stamp with microstructures. In the end, different complex 3D microchannels are achieved on various polymer wafers by transferring the pattern from the stamp. Ferreira et al. have recently developed a fast alternative to soft lithography to manufacture OOCs based on PDMS with integrated microactuators. The novel protocol decreases the complexity and number of steps, and is more time and cost-efficient compared to complex multilayered microfluidic devices [41].

Replica molding represents the technology in which a patterned silicon mold is employed, followed by the pouring step of a liquid polymer (usually PDMS) onto the mold for thermal crosslinking [42]. Later, the PDMS instrument is removed from the substrate and fixed on a clean, smooth wafer (for instance glass) to achieve a microfluidic chip with microfluidic networks.

The microcontact-printing technology is very similar to the replica-molding technique [36]. It is differentiating only by the supplementary steps further used to manipulate the pattern of cultured cells by printing the PDMS stamp on the wafer with biomolecules (such as proteins) in a designed pattern so that the cells on the membrane can be modeled as well by adjusting the pattern of the printed proteins [43,44]. However, even though microfluidic devices have been successfully fabricated by lithography or related techniques, the procedures are still costly and time-consuming. Additionally, these procedures are able only to produce the microchip itself, while other components such as microtissues, mechanical stimuli, or result detectors need to be separately produced.

**Figure 1:**
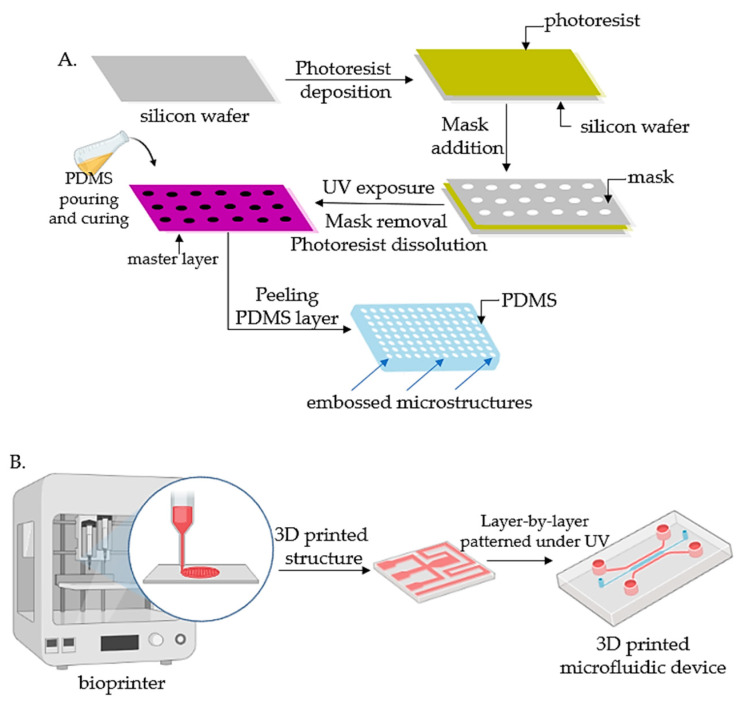
Fabrication technologies for OOCs: (**A**) The fabrication of micropatterned slabs of PDMS through photolithography; (**B**) Schematic 3D-printing process for the fabrication of microfluidic devices.

Recent advances in lithography-based fabrication techniques have emerged in high-throughput technologies with a standardized format, being more user-friendly and affordable for large preclinical research studies. The OrganoPlate system (Mimetas, Leiden, The Netherlands) is a commercial compact microfluidic device that can process 96 independent cultures using a standard microtiter plate. The microdevice replaces the inner membrane with a gel-media boundary for transepithelial transportation showing good results for the investigation of fluidic diffusion, tumor-cell invasion, and aggregation and toxicity assays [2]. The main technologies used for the microfabrication of OOCs are shown in Figure 1.

Injection molding allows fast replication of polymeric (micro)structures with great surface quality. Generally, the injection-molding process requires a few basic steps: (a) the material is melted and the molds are compressed together, and (b) the material is injected into the mold and cooled down prior to removal [34]. Although it has the advantage of large-scale production of OOCs, the fabrication process requires the careful adjustment of multiple parameters such as injection pressure, injection speed, melting temperature, etc. [45], to ensure high-quality production. Thermoplastic materials such as polystyrol [46] were used for the fabrication of liver-on-a-chip microdevices. One of the main disadvantages of this technique refers to the limited area of materials that can be used, as most of the polymers show thermal shrinkage during the fabrication [45]. However, the injection-molding process is reducing the time of fabrication and the final costs and therefore is predominantly used for the production of the commercially available elements of OOCs.

Laser-ablation methods, such as micromachining or computer numerical-control (CNC) micromachining, are surpassing the limitations of manual control during the fabrication of OOC microdevices. Laser micromachines use a laser to engrave the OOC device, and the process is applicable to a wide range of materials such as metals, glass or polymers. Shaegh et al. [47] designed a rapid prototyping method to produce microfluidic chips from thermoplastics with patterned microvalves combining laser ablation and thermal-fusing bonding. In this study, a CO_2_-assisted laser micromachine was used to pattern and cut PMMA layers covered with polyurethane film in order to generate a gas-actuated microvalve for microfluidic lab-on-a-chip applications. CNC micromachining is a fully automated manufacturing process in which the machines are operated via numerical control, wherein a computer software dictates the shape of the desired object. The laser-processing techniques have the advantage of rapidly creating precise and complex geometries of microdevices. Moreover, the laser-ablation techniques may be combined with the previously discussed methods for rapid prototyping or master models in order to achieve the desired microfluidic system, but require high technical knowledge for operating and expensive machinery to perform OOC fabrication [34].

#### 2.1.2. Bioprinting

Bioprinting technology has emerged in the last years as a versatile tool based on layer-by-layer addition that can be easily used for tumor-chip manufacturing [48,49,50]. A significant advantage is related to a wider range of materials and cells that can be printed simultaneously onto a substrate of cell-compatible biomaterials to construct 3D composite blocks with good spatial resolution and reproducibility [51,52]. Bioprinting also comprises a large group of procedures that can be divided into fused-deposition modeling (FDM), stereolithography, inkjet printing, and laser-assisted bioprinting [53]. Bioprinting procedures are appealing due to numerous advantages that accommodate the tumor-chip fabrication. Bioprinting technology can reconstruct the diverse TME and the 3D nature of the tumor by using bioinks that form cell conglomerations that can include various cell types, such as tumor-related fibroblasts, immune cells, and endothelial cells, to create vascular systems [54]. In addition, layer-by-layer bioprinting enables the formation of the biomimetic microenvironment for a heterogenous supply of biologically important proteins and growth factors that are essential to managing cancer-cell signaling, growth, and invasion [55]. Furthermore, the bioprinting technique allows for the printing of cells quickly and precisely in microfluidic chips, pattern vasculature, and model biological barriers. Vascularization channels play a significant role as they are vital to preserve tissue activities or to differentiate tissue parts. The tumor vasculature is much distinct from the blood vessels of healthy tissues, showing alterations in heterogeneity, multidirectional blood flow, permeability, and unordered distribution all over the diseased cells [56]. Proper vascularization of specific cancer-type cells has constantly been questioned in the production of functional in vitro tumor-cell cultures. Bioprinting technology manages to overcome these difficulties, showing the ability to replicate the abnormalities encountered in tumor tissue by building miniaturized pathophysiological models and offering control of features at the same size scale of living cells [57,58,59]. The main polymers used as bioinks for OOCs by bioprinting are summarized in Table 1.

### 2.2. Material Requirements for the Fabrication of Organ-on-Chips

Many biomaterials and fabrication methods have been recently developed to meet the OOCs’ functions. While designing the OOC microdevice, the organ or tissue characteristics and behavior must be considered to accurately simulate the in vivo motions, cell proliferation, or drug responses. Considering the organ characteristics aimed to be mimicked, different types of OOCs can be created.

However, although each type of OOCs may function differently, the main components remain the same. The OOC system is generally equipped with two compartments: first, the blood vessel compartment, which contains the endothelial cells; and secondly, the organ compartment, in which the cells of the investigated organ are introduced. These compartments are usually separated by a porous polymeric membrane able to provide cell communication between the two compartments of the device. Thus, the polymeric membranes play an essential role in the successful functioning of the OOC system, acting as interfaces with selective permeability for cell adhesion and cell separation.

The culture medium included in OOCs system may be artificially produced or naturally derived from living organs. The natural medium mainly contains biological fluids such as blood, plasma, etc., and organ fragments. The artificial environment is fabricated by involving nutrients that mimic the physiological conditions, such as salts, oxygen and CO_2_ gasses, and blood substitutes. Thus, the cell-culture medium will provide a continuous supply of nutrients and the porous polymeric membrane will deliver the cells of interest that are cultured on the other side of the membrane. In addition to the nutrient-rich fluid perfusion and oxygen ventilation from the blood-vessel compartment, in the organ compartment, mechanical forces can be applied to the polymeric membrane to simulate the peristalsis respiratory motions from the in vivo organs.

Despite the significant progress in material science, only a few materials can recapitulate the physiological conditions of living organ testing. First and foremost, the materials involved in the construction of the OOCs microdevices should ensure the necessary biocompatibility to provide a biologically safe and nontoxic environment that allows cell migration without generating any inflammatory response or exacerbated immunogenicity. Polymeric materials are desirable because of their great structural similarity to the ECM.

Besides biocompatibility, an essential requirement for the polymeric membrane that separates the OOCs compartments is the similarity with the ECM in order to support the cell attachment and diffusion through its pores from one side of the interface to the other compartment. Proteins such as collagen, fibronectin, and vitronectin are ideal candidates as they are involved in biological processes and play an important role in the cell-adhesion mechanism. Moreover, it was shown that the surface roughness of the membrane could affect cell behavior. In the case of synthetic polymers such as PMMA layers, it was observed that the cell adhesion and spreading of vascular cells improved with higher surface roughness, but proliferation was not affected, and it was indicated that the cell adhesion is dependent on the protein adsorption [70]. In the case of PDMS, surface roughness and surface energy play a key role in the cell-attachment process. It was observed that higher surface energy promotes the formation of stronger cell–ligand bonding, leading to improved cell growth and proliferation. However, the cell-membrane interaction drastically decreases above critical surface energy and critical roughness ratio. Thus, the study on the cellular behavior of HeLa and MDA MB 231 cells on a rough PDMS surface revealed that the optimum conditions for cell adhesion, growth, and proliferation are obtained at moderate surface energy and intermediate roughness ratios [71].

Additionally, the hydrophilicity of the polymeric membrane represents an important factor in determining the protein adsorption and, therefore, cell adhesion. It was observed that the hydrophobicity of the polymeric membrane could cause the absorption of small nonspecific molecules such as drugs or biomolecules from the cellular media, misleading the experimental results [72]. For this reason, most synthetic polymers have been submitted to chemical or physicochemical surface modifications to increase the hydrophilicity of the materials by enhancing their wettability properties. PMMA structures have been modified with different hydrophilic functionalities such as aminated polyethyleneglycol [73] or submitted to oxygen plasma treatment for the activation of the PMMA surface [74] to control the cell adhesion. The cellular adhesion is strongly determined by the surface wettability and roughness of the polymer, while the cell attachment is influenced by the cell type [75]. Premnath and colleagues [76] developed a simple approach to laser-modify the surface of a silicon chip to adjust cell adhesion and proliferation, and showed that the cervical cancer cells’ behavior is modulated to migrate onto untreated sites. Transparent polyethersulfone (PES) membranes have proven to be with optimized morphology, and have showed improved adhesion and cell viability compared to the commercial hydrophilic polytetrafluoroethylene (PTFE) [77]. Moreover, the increase in hydrophilicity of the polymeric layers enhanced the cell adhesion as well as the cell-adhesive proteins. It is worth mentioning that hydrophilicity/hydrophobicity balance can also control the essential nutrient diffusion through the polymeric membrane from the OOCs compartments and generally, water contact-angle measurements are performed to determine the wettability properties of the polymers (Table 2).

As the surface properties such as surface roughness and wettability influence the first stages of cell adhesion and migration, the mechanical properties of the polymeric membrane are modulated by the material’s stiffness and influence the later stages of cell growth. Investigations such as tensile strength, compressive stress, and wettability are generally achieved to determine if the polymer properties are suitable for the application. Thus, the mechanical performances of the interface membrane from the OOCs should be chosen accordingly to the native organ characteristics and to mimic the occurring physiological stimuli present in the replicated environment. Moreover, the porosity of the membrane layer represents an essential feature as it provides cell communication between the chip compartments and allows nutrient transportation to the cells. Pore size and shape also determine the space available for the cells to migrate, and small pores mainly reduce the cell adhesion, but increase the barrier function of cells.

### 2.3. Polymers for Organ-on-Chips

Materials used to manufacture OOCs play a crucial role as they may directly interact with the biological fluids and cellular system, affecting the experiment results. Although several types of polymeric structures have been employed, only a few polymers exhibit the required specifications needed to fabricate the OOCs, and both synthetic polymers and biopolymers have been investigated.

In the last few years, synthetic and natural polymers have been used for OOC applications as their performances depend on the polymer structure, porosity, transparency, flexibility, stiffness, etc. Synthetic polymers such as polydimethylsiloxane (PDMS) [101], polycarbonate (PC) [102], polyethylenetereftalate (PET) [103], aliphatic polyesters, polyurethanes [104], etc., have been employed for the preparation of porous-compartment-separation membranes due to their easily adjusting porosity, surface roughness, and mechanical properties. Exhibiting improved biocompatibility, natural polymers such as collagen, gelatin, or polysaccharides have attracted significant attention lately due to their faithful replication of the native tissues with channel interconnections that allow the perfusion of oxygen and nutrients to simulate the natural behaviors of cell differentiation, spreading, and adhesion along the separating membrane [105]. However, natural polymers show batch-to-batch composition variability and lower mechanical properties, thus affecting the reproducibility of the experiments. However, each employed biomaterial still exhibits weaknesses to achieve the optimal OOC performances.

Polydimethylsiloxane (PDMS) is an elastomeric polymer and one of the most employed synthetic materials for organ-chip production, as it exhibits optical transparency, biocompatibility, gas-permeation properties, flexibility, and allows permanent microscopic observation of 3D cell structures for real-time assessment of tumor behavior and response to therapy [101,106,107]. Microfluidic systems made from PDMS offer a much closer physiological microenvironment to that of the living TME from a biomechanical point, exhibiting porosity and significantly lower stiffness. However, PDMS exhibits an important limitation in chemical screening due to its hydrophobicity, which leads to nonspecific absorption of small molecules such as drugs or pharmaceutical compounds [108,109]. Nevertheless, the limitations of PDMS materials have been overcome through surface modification treatments with another polymer that enhances the hydrophilicity of the PDMS-based materials to facilitate cellular adhesion and migration in microfluidic compartments. One of the most commonly employed surface-modification approaches consists of oxygen-plasma treatment by converting some hydrophobic methyl groups into hydroxyl functionalities via oxidation. It was reported that the hydrophilicity of the oxidized PDMS after plasma treatment can increase by almost 30° [110].

Moreover, the wettability behavior of PDMS materials can be improved by coating the polymer layers with other hydrophilic polymers. The proteins from ECM composition are generally used to achieve the natural moiety of PDMS to ensure cell attachment and proliferation. ECM proteins, such as collagen and fibronectin, create covalent bonding onto the PDMS surface and thus facilitate cell adhesion by modifying the surface roughness of the synthetic polymer [111]. Although the hydrophilicity and biocompatibility are significantly increased this way, dissociation processes during protein coating are associated with this approach [112]. Other biopolymers, such as gelatin [113], fibronectin [114], or hydrophilic synthetic polymers such as polydopamine [115,116] and polyethylene glycol [117] have been successfully employed for modifying the surface roughness of PDMS and managed to enhance the cellular adhesion for short-term cellular culture.

Polycarbonate (PC) structures are commonly used polymeric materials for the OOCs fabrication and as porous membranes for Transwell^®^ inserts. The PC structures are hydrophobic, exhibit high transparency, and are inert and stable under biological processes [118]. Similar to PDMS, the surface of PC is commonly modified by gas plasma treatment or protein coating [119]. However, PC structures exhibit higher rigidity than PDMS. They are not suitable for stretchable membranes or soft substrates.

Other thermoplastics are tested, such as polyolefins [103], styrene-ethylene butylene styrene (SEBS) [120], polyurethanes [121], and copolymers [122], but there is still a demand for novel materials in this domain.

Polyolefins such as cyclic olefin polymers (COP) and cyclic olefin copolymers (COC) are thermoplastic materials that have also been employed for the manufacturing of microfluidic devices, being less gas-permeable than PDMS. COP and COC are lipophobic materials and do not exhibit unspecific adsorption of small molecules, allowing their use in drug-development and diffusion experiments [122]. Moreover, they exhibit optical transparency, good chemical resistance to polar solvents, thermal resistance, and reproductible mass production.

Polymethylmethacrylate (PMMA) is another frequently used material as a substrate [123,124]. Kang and coworkers [125] demonstrated that PMMA chemically attached to porous orbitally etched polyethylene terephthalate (PTFE) membranes is impermeable to small hydrophobic compounds and more consistent results concerning the anticancer vincristine drug cytotoxicity on human lung adenocarcinoma cells cultured in PDMS-based chip. Thus, PMMA shows promising features for tumor chip microdevices fabrication as the small molecules cannot infiltrate the material.

Aliphatic polyesters such as polylactic acid (PLA) and poly (ε-caprolactone) (PCL) have been used to mimic the in vitro blood–brain barrier, functioning as membranes in OOCs microdevices. Both polymers are biodegradable and hydrophobic. Although their surface can be chemically modified to enhance the wettability and cell proliferation, these polymers have been sparsely employed as they tend to degrade under biological processes releasing acidic degradation products that could affect the cellular medium [123].

To fulfill the requirements of mechanical actuatable OOCs, the employed materials need to be optically transparent, flexible, easily moldable, and nonabsorptive to drugs and cell nutrients. Moreover, it is necessary to find sustainable alternative materials. At present, the manufacturing process of OOCs and experimental validation remain high-priced so that less-expensive and reusable materials should be employed.

Gelatin is an animal protein obtained by collagen hydrolysis, and is very much employed in drug-delivery and tissue-engineering applications [126] due to its ability in drug transportation and cell-growth promotion [127]. Gelatin exhibits biocompatibility and flexibility, and forms complexes with proteins, growth-factor nucleotides, and biopolymers, and can be shaped in colorless gels [128]. Additionally, photo-crosslinked gelatin methacrylate (GelMa) is highly used in tissue engineering and shows interest in OOC formation [129,130].

Collagen-based ECM gel (known as Matrigel) is a commercial product that contains ECM hydrogel made of tumor-derived basement membrane proteins employed for cell culture [131]. Matrigel gives structural and signal-transduction functions. Tumor cells exhibit aggressive behavior in Matrigel medium, and it is frequently used to assess the malignancy of cancer cells and observe the mechanism of tumor growth [132].

Bacterial cellulose paper has also attracted interest for tumor-chip applications as it has good biocompatibility, and it is a naturally derived polymer that is densely vascularized through nanofibers [133,134]. Recent advances in bioprinting are multiplying the paths for creating complex perfused systems, and the discovery of new materials that accomplish the full requirements of native living cells is still under extensive development.

## 3. Kidney-on-Chip

The kidney is the second major target of drugs and chemicals after the liver, receiving 25% of the cardiac output and being responsible for drug and metabolite excretion and fluid–electrolyte balance. Thus, exposure of the kidney to high concentrations of xenobiotics and drugs metabolites leads to drug-induced nephrotoxicity, and furthermore to (i) acute kidney injury (AKI), which is often associated with increased morbidity and mortality; (ii) chronic kidney disease (CKD); and (iii) end-stage renal disease (ESRD) in the general population [135]. The physiological function of the kidney is supported by a very complex architecture of the renal tissue that consists of a 3D distribution of more than ten cell types within an ECM and an elaborated vasculature system with high blood flow [2]. The kidneys are responsible for maintaining the balance of the body fluids and also discarding metabolites regulating filtration, reabsorption, and secretion processes through the nephron, the kidney’s functional unit. The nephron consists of (i) the renal corpuscle, which includes the Bowman’s space, encapsulating the capillaries (glomerulus); (ii) the proximal renal tubule, which reabsorbs sodium chloride and sodium bicarbonate, completes the reabsorption of glucose, and also is the unique site of amino-acid and anion transport; and finally (iii) the renal distal convoluted tubule, which plays a critical role in sodium, potassium, and divalent cation homeostasis [136,137].

The mechanisms through which various drugs are nephrotoxic are diverse and can be selective or non-selective, depending on whether they injure a specific type or multiple types of cells. Moreover, the nephrotoxic effect can be direct, many drugs having an affinity for specific transporters throughout the nephron and causing cell death in the corresponding segments through a variety of mechanisms, or indirect, secondary to the osmotic effect and hemodynamic changes (ex: iodinated contrast agents), drug-induced nephrolithiasis or ischemia.

### 3.1. In Vitro 2D/3D Models for Kidney Disease Modeling

Current approaches to further testing drugs before entering clinical trials to assess nephrotoxicity are using in vitro models, such as 2D, 3D, and microfluidic models—kidney-on-chip and the more recently introduced approaches based on stem cells [3].

The majority of the 2D models use cell lines distributed in tight monolayers, such as porcine LLC-PK1 cells and Madin–Darby canine kidneys. Human cells such as the renal tubular cell line HK-2 have also been used in vitro, albeit with limited applicability due to their poor expression of the SLC2 transporter family. Moreover, due to the kidney’s complex structure sustaining its function, the 2D models (despite being the gold standard for a long time now) are a poor representation of the in vivo environment, including low expression of OATs and OAT2 transporters and poor apical-basal polarization [8]; 3D in vitro models known as organoids, are challenging to develop and are not currently validated for prediction of drugs nephrotoxicity. Most of them mimic only a part of the nephron (glomerulus or tubules) and are tested for a limited number of drugs. One of the challenges in recreating an in vitro nephron model is to step forward from traditional 2D models characterized by rather static conditions to mimic the dynamic conditions such as blood and urinary flow, exposing tubular epithelial cells to fluid shear stress facilitating transepithelial osmotic gradient [138]. For kidney-tissue engineering, the most challenging part is recreating the vascular network. Xuan Mu et al. tried to mimic a nephron by combining fibrillogenesis and liquid molding to build a 3D vascular network in the hydrogel. They succeeded in obtaining a cytocompatible, mechanically stable vascular network that could mimic passive diffusion of organic molecules and is showing promise in simulating physiological functions based on active mass transfer in hydrogels. This would benefit not only the drug screening for nephrotoxicity but also other vasculature-rich organ-related research [139].

### 3.2. Kidney-on-Chip Models

**Figure 2:**
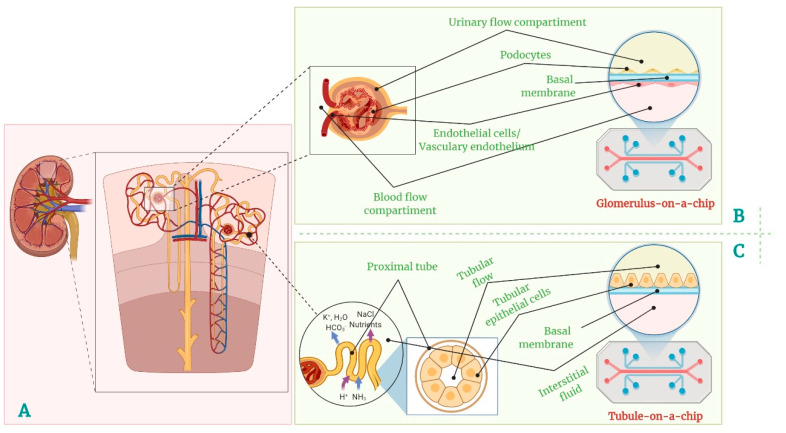
Complex kidney-on-a-chip for personalized medicine: (**A**) kidney and nephron, (**B**) glomerulus-on-a-chip, and (**C**) tubule-on-a-chip.

In this view, the kidney-on-chip approach tries to mimic the dynamic, flowing environment more closely and consists of a 2D/3D cellular model developed in a microfluidic device. This whole system finally consists of a 3D architecture built up by renal cells grown on an ECM interface or next to perfusable microchannels where the media and/or other body fluids such as blood or urine can flow across the cell surface [140] (Figure 2). Using these models, studies have shown that fluid shear stress (FSS) has a major influence on renal tubular cells’ phenotype [141], hence their importance in nephrotoxicity and other renal-function-related studies. FSS can induce inflammation through immune-cell-mediated activation and monocyte adhesion [142]; it increases the expression of certain genes such as ABCG2, RBP4 (a marker for tubular function loss) [143], CYP1A1, and SLC47A1, affects the cytoskeleton organization, and upregulates the formation of tight junctions [144]. Consequently, kidney-on-chip models are designed for high-throughput screening of drug toxicity by delivering essential output data regarding the glomerular filtration processes, the drugs pharmacokinetics with impact on drugs validation, and relevant dose determination. In this view, several types of kidney-on-chip models have been developed targeting different segments of the renal unit.

#### 3.2.1. Glomerulus-on-Chip

The glomerulus is the filtering unit of the kidney, consisting of a capillary network and podocytes, highly differentiated epithelial cells that are responsible for the actual filtration process of the blood. Considering this, there is an obvious interest in developing a functional system recapitulating the glomerular function to support preclinical stages of new drug development or to sustain research in the kidney disease field, including (but not limited to) kidney neoplasms. Despite the efforts made in the past few years to recreate a glomerulus-on-chip system, the main challenge in developing an in vitro glomerulus model is the lack of functional human podocytes. Various studies have been performed to create a glomerulus-on-chip model by obtaining human-induced pluripotent stem-cell (iPSCs)-derived podocytes and placing them in a microfluidic device together with endothelial cells (primary or secondary iPSC-derived). These microfluidic devices were designed to create the two compartments separated by a porous membrane. Musah et al. showed that podocytes extended their processes through the membrane when exposed to constant flow and mechanical strain, and selective filtration was proven by the presence of excreted inulin and retention of albumin on each side of the membrane [145]. Roye et al. also tried to build a glomerulus-on-chip with hPSC-derived podocyte and endothelial cells, and succeeded in obtaining essential functionality and structure [146]. Both models were carried out to study the effect of adryamicin as a chemotherapeutic agent, and showed that the treatment induced podocyte detachment and endothelial-barrier disruption, leading to albuminuria. This glomerulus-on-chip model could be a promising start for an in vitro study of proteinuria and glomerular kidney disease and chemotherapeutic drug-nephrotoxicity assessment. Moreover, in 2017 Wang et al. developed a glomerulus-on-chip model using rat cells to create a diabetic nephropathy model that also served to assess the hyperglycemic pathological response [147].

#### 3.2.2. Tubule-on-Chip

Most kidney-on-chip studies use proximal tubule cells, as this part of the nephron is the primary site of drug clearance and a critical target for drug-induced nephrotoxicity. Therefore, the in vitro reproduction of proximal tubule function is of major interest in the preclinical assessment of candidate compounds. For this, hollow fibers are used as scaffolds to design a tubular model that resembles the structure of the proximal tubule. Such models were developed by coating the hollow fibers with a hydrogel that served as ECM for human proximal-tube endothelial cells (hPTECs) [148]. This approach enabled the observation of the secretory clearance of albumin-bound uremic toxins and albumin reabsorption [149]. Other studies developed a proximal tubule-on-chip using a polyester membrane to split the main compartment of the device into a luminal-like channel and a basal interstitial-like channel that were populated with PTECs seeded on an ECM coating [90]. This study reports significant changes in PTEC physiology and morphology, such as polarization, display of columnar shape, primary cilia, and transporters due to the simulated FSS induced within the system, and highlights the impossibility to achieve all these under static-culture conditions. These observations were also confirmed by other studies [150,151]. Another strategy in achieving proximal tubule-on-chip systems is bioprinting of tubular 3D architectures [152]. Homan et al. used this technique to obtain functional proximal tubule-on-chips by growing PTECs on a fibrinogen ECM coating the lumen of a structure printed with Pluronic ink [153].

One of the first kidney-on-chip models for nephrotoxicity screening used primary renal tubular cells to assess cisplatin toxicity, and the results most resembled in vivo models [154]. Other nephrotoxicity studies performed on kidney-on-chip models include the study of ifosfamide and acrolein nephrotoxicity assessment performed by Le Clerc et al. [155], the gentamicin kinetics study using MDCK on a porous membrane coated by fibronectin [156], and the toxicological assessment of polymyxins and their nephrotoxic potential on a 3D MPS human model [151].

Lastly, one interesting approach in nephrotoxicity testing is using versatile high-throughput screening platforms such as the OrganoPlate™ system powered by Mimetas for robust and reproducible results in preclinical studies. In this view, several studies report the expression and function of renal transporters and cell-polarization response to cisplatin [157] by modeling the proximal tubule functions in independent chips placed in a standard microtiter plate [158].

### 3.3. Multiorgan-on-Chip Models That Include Kidney-on-Chip

Taking the use of OOC models even to the next level, recent studies report the development of multiorgan-on-chip or body-on-chip models as platforms enabling the screening of multiorgan toxicity. In particular, integrating a kidney-on-chip device into a multiorgan system would bring valuable insights regarding secondary toxicity of drugs beyond systemic toxicity and the inflammatory response. Maschmayer et al. created a four tissue coculture (skin, small intestine, kidney, and liver), with 28 days of reproducible capacity, on a microphysiological four-organ-chip model, which is a promising line of research for pharmacodynamic and pharmacokinetic drug parameters and toxic profile [159]. Chang et al. used a combination of liver and kidney cells on a chip platform to study the nephrotoxic effect of aristolochic acid, a nephrotoxic agent that first needs to be activated in the liver [160].

## 4. Bladder-on-Chip

The urinary bladder is a hollow organ located in the lower abdomen that acts as a reservoir for the temporary storage of urine received from kidneys, further expelled during micturition through the urethra [161]. As a structure, the urinary bladder is composed primarily of smooth muscle, collagen, and elastin [162]. Microscopically, the urinary bladder has a stratified architecture, organized in the following layers: lining epithelium (urothelium), lamina propria, muscularis propria, and serosa, the urothelium acting therefore as a barrier that is exposed to potential carcinogens [163]. Among urinary bladder diseases, cystitis and cancer are the most common; thus, the existing OOC platforms developed until now address these pathologies, with a significantly limited number of published studies available that show the OOC platforms as tools for bladder research are yet to be explored. The use of bladder-on-chip for disease modeling could pave the way for a better knowledge of the disease physiopathology and could advance research by accelerating the discovery of novel drugs, as well as by significantly improving drug-efficacy studies.

### 4.1. Modeling Bladder Cancer

Bladder cancer (BC) is the most common cancer of the urinary system and ranks globally as the 10th most common type of cancer, a pathology that affects men more than women [164,165]. BC is defined as a carcinoma of the urothelial cells and can be divided by the tumor depth of bladder-wall invasion in non-muscle-invasive BC (NMIBC), muscle-invasive BC (MIBC), and metastatic BC, subtypes that are characterized by different molecular signatures, BC being one of the most frequently mutated human cancers [166,167]. While the approval of immunotherapy for both NMIBC and MIBC, as well as targeted therapy (erdafitinib) and antibody-drug conjugate therapy (enfortumab vedotin) significantly improved the therapeutical management of BC, the alarming statistics regarding the disease recurrence and overall survival of BC patients highlight the emerging need for developing better preclinical study models [168]. At the moment, the most intensively used preclinical models for BC research are in vitro 2D cell cultures (cell lines, conditionally reprogrammed cell cultures) and 3D organoids, and in vivo carcinogen-induced mouse models, genetically engineered mouse models, and patient-derived xenograft. However, each preclinical model presents unique features, together with different disadvantages such as failure to mimic the 3D tumor microarchitecture, microenvironment, and tumor heterogeneity; and lack of immune system, thus failing to ease the transition between preclinical models and clinics [169,170,171,172]. For example, a highly neglected aspect of many preclinical models is the presence of the tumor microenvironment consisting of malignant and nonmalignant cells (cancer-associated fibroblasts, cancer-associated immune cells), ECM, and blood vessels, an entity that orchestrates cancer progression and modulates therapeutic response [20].

In this view, Liu and colleagues [173] aimed to reconstruct the bladder microenvironment by coculturing four types of cells into a two-layer microfluidic device made of a PDMS piece and a glass slide. The fabricated microfluidic device connected to perfusion equipment featured four indirectly connected cell-culture chambers (BC cells, fibroblasts, macrophages, endothelial cells), ECM channel units (Matrigel), and culture-medium channels. Therefore, using this microfluidic device, four types of cells were allowed to simultaneously interact through soluble biological factors and metabolites that proved to diffuse through the ECM channel units between cell-culture chambers, in a dynamic setup provided by continuous medium perfusion. The validated microfluidic system proved to be a good platform for cell-motility patterns and phenotypic alteration of stromal cells, as well by generation of reticular structures based on BC cells, and opened the perspective of OOC implementation for precision medicine, as BC cells treated with different clinical neo-adjuvant chemotherapy showed different treatment responses, revealing the drug sensitivity of tumor cells in this experimental setup. In another study, a microfluidic chip was designed to coculture BC cells and fibroblasts and further analyze changes in mitochondrial-related protein expression of these cells and their characteristics of energy metabolism [174].

Therefore, to investigate tumor metabolism, the microfluidic chip fabricated was composed of four cell-culture pools, two for BC cells and two for fibroblasts, interconnected through two ECM microchannels (Matrigel) and two microchannels with the outside, which were continuously fed with cell-culture medium injected through the peripheral perfusion channel. By comparing the coculture system with individual cultures of BC cells and fibroblasts, significant differences in lactic-acid concentration and mitochondrial-related protein expression were observed; results that revealed that cells conduct glycolysis more efficiently under coculture conditions, and show enhanced overall mitochondrial activity and protein expression, highlighting the importance of using OOC in bladder tumor energy-metabolism studies. Finally, Lee et al. [175] designed a simple 3D microfluidic device based on PDMS and Matrigel to culture MIBC cells and NMIBC cells, for modeling metastasis. The study showed an increased expression of CD44 and RT4 after 2 weeks of culture in MIBC cells as compared with NMIBC cells, associated with a significant increase of MMP-9 gelatin degradation, showing that the OOC system could be further employed for migration and metastasis studies.

### 4.2. Modeling Urinary Tract Infections

On the other hand, urinary tract infections (UTI) are among the most common bacterial infections, divided into uncomplicated and complicated infections, isolated infections of the bladder being referred to as cystitis and treated subsequently with antibiotics [176]. However, despite completing the antibiotic-based treatment regimen, cystitis is characterized by a high recurrence frequency, which involves readministration of antibiotics [177]. UTI is most frequently caused by uropathogenic *E. coli* (UPEC) bacteria, which underlies more than 80% of the diagnosed cases [178]. Once entering the urinary bladder, the *E. coli* can float freely in the urine and be eliminated through micturition or form intracellular bacterial communities (IBC) that the bladder struggles to clear [179]. Therefore, to reveal the insights of UPEC infection and IBC formation, Sharma and colleagues [180] used a commercially available OOC purchased from Emulate to model UPEC infection and mimic the interface between the blood vessels and the tissue layers of the human bladder. To reconstruct the bladder native architecture, human bladder microvascular endothelial cells were cocultured with human bladder epithelial cells and exposed dynamically to urine and nutritive cell-culture media, while using the application and release of linear strain to recreate micturition. By infecting the epithelial layer underflow with UPEC and monitoring OOC by microscopy, the bacteria motility, interaction with cells, and IBC formation could be monitored. Moreover, the addition of human-blood isolated neutrophils into the OOC system revealed that their diapedesis to sites of infection on the epithelial side can lead to the formation of neutrophil swarms and neutrophil extracellular traps (NETs), and that IBCs offer substantial protection to bacteria from antibiotic clearance. Administration of antibiotics in the developed bladder-on-a-chip model through urine revealed their potential to kill bacteria floating freely in the urine much faster than bacteria residing in bladder cells, as well as the increased resistance of IBCs to treatment, aspect that highlight the importance of completely eradicating IBCs to avoid infection recurrence. No doubt, this complex study shows the potential of the bladder-on-chip as relevant platforms for modeling infections, as well as their use for drug screening of multiple antibiotics or novel drugs in a physiologically relevant manner.

## 5. Prostate-on-Chip

**Figure 3:**
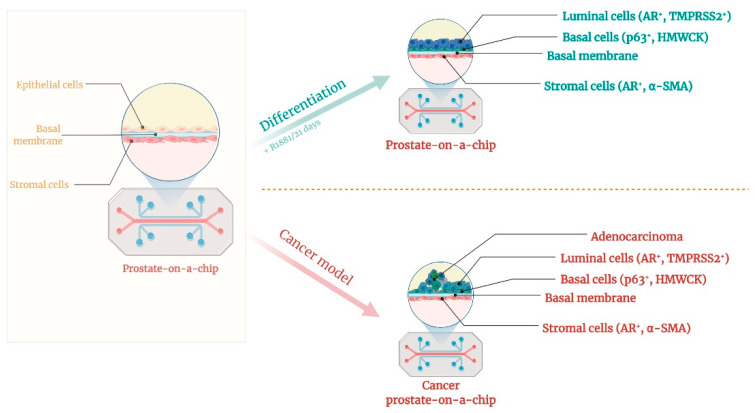
Prostate-on-chip.

The prostate is an exocrine gland that secretes sperm-nourishing and protective fluid. It is located beneath the urinary bladder and part of the male reproductive system [181]. The prostate-tissue architecture consists of ducts and acini lined with glandular or secretory cells on top of basal cells and surrounded by a fibromuscular stroma [182,183]. Consequently, the prostate displays two major components: one is the epithelium, containing neuroendocrine cells and basal cells that express integrins and hold the differentiation potential toward luminal (secretory) cells that will express androgen receptor protein [184,185]; the other component of the prostate tissue is the stroma, which is separated from the epithelium by a basement membrane. Prostate cancer is the most common cancer diagnosed worldwide in men and is characterized by molecular changes caused by genetic and epigenetic modifications leading to malignant transformation of the cells [186,187]. Androgens regulate prostate development and cell differentiation from embryonic development to adults, and thus, a better understanding of early interactions between prostate cells leading to development would be essential in unraveling the mechanisms underlying prostate cancer [185]. Many studies in this field have been developed, starting with 2D conventional cultures and 3D models, and ending with versatile proposals of prostate-on-chip systems. Likewise, for other previously discussed pathologies, in prostate cancer, 2D cell cultures fail to provide the proper tissue-level complexity, not only in their limitation to one cell type but also due to their missing crucial TME aspects, as well as FSS simulation [188]. The schematic representation of the prostate-on-chip model is presented in Figure 3.

On the other hand, animal models that do display the in vivo tissue-level complexity fail to mimic human anatomy or physiology [189]. To address this issue, various engineered microfluidic devices have been proposed for concomitant multiple cell-culture types. For example, Picollet-D’hahan, N. et al. used polyelectrolyte nanofilms to create a model of prostate epithelial cancer cells (PC3) and normal prostate epithelial cells (PNT-2) [190]. Furthermore, Jiang et al. [191] reported the development of a human prostate gland model for a better understanding of the epithelium/stroma interaction, both in terms of unrevealing the R1881-mediated epithelial cell (PrEC)-differentiation mechanism towards functional secretory cells and coculturing of these cells with prostate stromal cells (BHPrS1). For this, they designed a microfluidic device fabricated by soft-lithography techniques from Polydimethylsiloxane (PDMS), which incorporates a commercial polyester membrane of 0.8 μm-diameter pores, 1% porosity, and a thickness of 23 μm. The major advantage of this device is its ability to overcome the impossibility of establishing long-term 2D static cocultures of these cell types due to their different culture-media requirements [191]. Moreover, this device was able to sustain an in vivo-like behavior of the cells that displayed groups forming gland-like buds.

## 6. Benefits and Challenges of OOCs/TOCs in Urology

**Figure 4:**
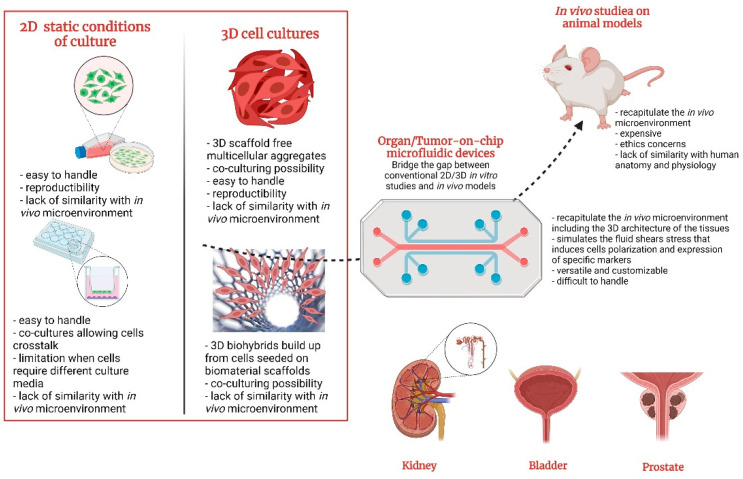
Schematic representation of the available in vitro and in vivo models for the urology-associated pathology study, highlighting their advantages and disadvantages and revealing the potential key role of the organ/tumor-on-chip devices to bridge the gap between conventional 2D/3D culture systems and animal models in preclinical studies.

Experimental research demonstrated that microfluidic OOC technology might function to screen recently developed anticancer compounds, cellular and nanotechnology-based treatments, improve therapy conditions, and establish the effects (or side effects) of combined therapies in in vivo-like TME (Figure 4).

Imitating organ-level pathophysiology found in vivo is the key factor in developing successful OOC models and requires clear efficacy validation. One of the main challenges in OOC fabrication and usage remains the straightforward optimization to functionally connect multiple organ systems in order to collect media from the output of the microdevice and feed it into another to accurately mimic the sequential adsorption, distribution, metabolism, and excretion of the compounds. The microfluidic technology allows perfusion of cell-culture medium through or across tissue structures and maintains biomechanical stimuli in a controlled manner (for instance, continuous, intermittent, or cyclic). Moreover, during an experiment, the easy access to cells or media for dosing, sampling, and analysis with small-molecule compounds (antibodies, hormones, drugs, etc.) offers an unquestionable advantage over the animal and other previous static models, including spheroids and organoid cultures. Previous studies already showed that TOCs mimic the native environment of cells, including 3D topology or physical stretch and strain, and they are able to maintain precise control over inter-and intra-organ flow rates, forming a miniaturized version of the human body. Additionally, bladder-on-chip models allow modeling of urinary infections by simulating *E. coli* bacterial infection of the urinary tract cells. These insights are crucial in the framework of studies that focus on in vivo processes such as angiogenesis, tumorigenesis, invasion, metastasis, and infection, enabling in vitro analysis of how local microenvironmental signals and chemical gradients influence these processes [192]. The existence of a perfused endothelium-lined capillarity endorses the superiority of OOCs over static models by providing precise control over cell-culture conditions and over platform pharmacokinetics and drug toxicity, respectively. The ability to adjust key parameters such as concentration gradients, cell patterning, tissue–organ interactions, and to replicate an organ-like mechanical microenvironment that exists in human living TME placed OOCs in the “Top 10 Emerging Technologies” at the World Economic Forum [193].

The multiple benefits of OOCs have made them indisputable alternatives for animal studies or 3D culture assays. However, clinical trials require time, and OOCs are still competing with animal testing to predict clinical responses. Moreover, OOCs are not considered as easy to be handled as conventional cell models. Although they allow long-term experiment and minimal user input, the technical robustness remains a challenge, as the compact and complex microfluidic devices are engaging multiple parameters that simultaneously run to achieve optimal functionality. Air-bubble formation or unintended infection risks at connection points are the most common factors that may lead to experiment failure. Shourabi et al. [194] recently designed an optimized integrated microfluidic gradient generator for mechanical stimulation and drug delivery by dynamic culturing of human lung cancer cells (A549 cell line) made from two PDMS-layer microfluidic chips with a porous membrane interfacing in between. The key feature of this system consists in two bubble trappers designed to remove the unwanted bubbles that could enhance the shear stress on cells. In this way, the concentration gradient generator’s performance is guaranteed, and precise control of fluid shear stress on cells is obtained. The results showed that the chip exhibits a high cell-viability rate (95%) and will be employed to study the toxicity effect of different concentrations of cisplatin on renal cells.

Microfluidic OOC microdevices can be also employed for studying the role of cancer stem cells (CSCs) in tumor initiation and cancer relapse [195]. As the chemotherapeutic regiments kill tumor cells but are ineffective on CSCs, the microfluidic OOCs have the potential to give insights on tumor cell–CSC interaction, as well as accelerating drug development to also target this subpopulation of cells [196]. By employing a biomimetic in vitro model such as OOCs for CSC research, various environmental parameters such as oxygen gradient, glucose, and fluid shear stress can be mimicked, offering valuable insights on CSCs’ multicellular interaction and drug-resistance mechanisms to conventional therapies [197].

However, there is still a place for developing new materials that facilitates the noninvasive assessment of drug effects and meets the industrial-scale production demands. Novel materials based on biopolymers have been developed in the last few years to overcome the main drawbacks of PDMS-based OOC microdevices, and significant attempts to produce scalable systems have been reported [198]. Therefore, as the technology has evolved in the last years and the introduction of commercial prefabricated tumor chips that are user-friendly and custom-made for culture and fluidic control is expanding [199], it will undoubtedly facilitate the possibilities of medical research for oncology treatments.

## 7. Conclusions and Future Considerations

Human OOCs/TOCs offer a promising alternative to conventional 2D/3D static cultures, and even to in vivo models that lack similarity with human anatomy and/or physiology. These microfluidic devices hold the potential to sustain relevant preclinical studies for developing new therapies, including anticancer drugs, and bring new insights for a better understanding of pathogenesis mechanisms. Replicating the physiological TME, TOC models are seen as promising and more realistic alternatives for investigating the metastasis, distribution, and mechanism of tumor propagation. Involving microfluidic technologies, OOC models can mimic the complexity of in vivo tumors and can be more accurately used to predict therapeutic efficacy and drug toxicity or side effects. Although OOCs closely mimic the native organ functions, one must consider that most of the cell cultures interact with polymeric substrates and porous polymeric membranes to replicate the physiological microenvironments. Thus, the polymer material properties such as surface roughness, wettability, and mechanical properties significantly affect cell adhesion and proliferation.

Moreover, size porosity and biodegradability influence cell migration and viability. While most of the studies present the use of common synthetic polymers beneficial for OOCs fabrication, other studies employ less common polymeric structures with improved cytocompatibility, wettability, or mechanical stability. Although there is a place for deeper investigations to optimize the materials’ properties and fabrication methods, the OOC/TOC models are adding necessary steps to personalized medicine for creating high-precision remedies with the possibility to use biopsy from the patient that could be expanded in vitro and evaluated for cost-effective screening of treatments that are particularly efficient for that patient.

## Figures and Tables

**Table 1:** The main polymers used as bioinks for OOCs by bioprinting.

Bioink Composition	Bioprinting Method	OOC Model	References
Collagen	extrusion	Lung, gut	[60,61]
Gelatin	extrusion	kidney	[62,63]
Methacrylate gelatin (GelMa)	extrusion	Vessel, liver	[64,65]
Alginate	extrusion	Heart	[66,67]
Cellulose	extrusion	tumor, liver	[68,69]
Polyethyleneglycol (PEG) Poly ε caprolactone (PCL)	inkjet, extrusion	Colon tumor	[59,65]

**Table 2:** The main polymers employed in the fabrication of membranes in OOCs microdevices.

Polymer	Chemical Structure	Contact Angle with Water	Young’s Modulus	Application
Polydimethylsiloxane (PDMS)	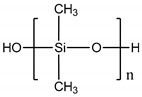	107° [78]	Variable from kPa to MPa	Cardiovascular [79] Kidney [80] Liver [81] Lung [82]
Poly (bisphenol-A- carbonate) (PC)	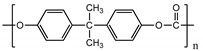	~85° [83]	~1.2 GPa [84]	Tumor vasculature [85] Colon and breast cancer [86]
Poly (ethylene terephthalate) (PET)	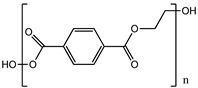	83° [87]	4.7 GPa [88]	Gut [89] Kidney [90] Liver [91]
Polylactic acid (PLA)	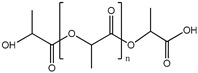	~75° [92]	3.1 GPa [93]	Endothelial barrier [94]
Poly (ε-caprolactone) (PCL)	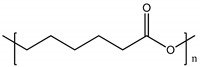	120° [95]	~400 MPa [96]	Blood-brain barrier [97]
Polytetrafluoroethylene (PTFE)	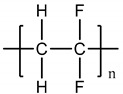	108° [98]	392 MPa [99]	Liver [100]

## Data Availability

Data are contained within the article.
